# The Preventive Effect of Oxytocin to Cisplatin-Induced Neurotoxicity: An Experimental Rat Model

**DOI:** 10.1155/2015/167235

**Published:** 2015-01-22

**Authors:** Tulay Akman, Levent Akman, Oytun Erbas, Mustafa Cosan Terek, Dilek Taskiran, Aydin Ozsaran

**Affiliations:** ^1^Division of Medical Oncology, Tepecik Education and Research Hospital, Gaziler Caddesi No. 468, Yenisehir, 35110 Izmir, Turkey; ^2^Department of Obstetrics and Gynecology, Faculty of Medicine, Ege University, Izmir, Turkey; ^3^Department of Physiology, Faculty of Medicine, Ege University, Izmir, Turkey

## Abstract

Peripheral neurotoxicity is a frequent dose-limiting side effect of the chemotherapeutic agent cisplatin. This study was conducted to investigate the preventive effect of oxytocin (OT) on cisplatin-induced neurotoxicity in rats. Forty-four adult female rats were included in the study. Thirty-six rats were administered intraperitoneally (i.p.) single dose cisplatin 10 mg/kg and divided in to 3 groups. The first group (*n* = 12) received saline i.p., whereas the second group (*n* = 12) and the third group (*n* = 12) were injected with 80 *µ*g/kg and 160 *µ*g/kg OT, respectively, for 10 days. The remaining 8 rats served as the control group. Electromyography (EMG) studies were recorded and blood samples were collected for the measurement of plasma lipid peroxidation (malondialdehyde; MDA), tumor necrosis factor (TNF)-*α*, and glutathione (GSH) levels. EMG findings revealed that compound muscle action potential amplitude was significantly decreased and distal latency was prolonged in the nontreated cisplatin-injected rats compared with the control group (*P* < 0.005). Also, nontreated cisplatin-injected rats showed significantly higher TNF-*α* and MDA levels and lower GSH level than control group. The administration of OT significantly ameliorated the EMG alterations, suppressed oxidative stress and inflammatory parameters, and increased antioxidative capacity. We suggest that oxytocin may have beneficial effects against cisplatin-induced neurotoxicity.

## 1. Introduction

Cisplatin (*cis*-diamminedichloroplatinum) is the first antineoplastic agent which has been used in cancer treatment. It is used for the treatment of various solid tumors such as lung, ovary, testis, bladder, head and neck, and cervical and endometrial cancers [1]. Autotoxicity, neurotoxicity, and nephrotoxicity are the dose-limiting side effects of cisplatin [2]. Cisplatin-induced peripheral neuropathy is a frequent adverse effect leading to a dose reduction or the early cessation of chemotherapy, thereby potentially impacting patient survival [3]. Peripheral neuropathy is characterized with decreased neural transmission rate, loss of vibration and position senses, tingling paresthesia, dysesthesia, loss of tendon reflexes, tremor, ataxia, and muscle weakness [4–6]. The side effects of the cisplatin on both human and animal nervous systems are demonstrated with electrophysiological and histopathological experiments [5, 7–9]. Oxidative stress, DNA damage, and inflammatory cytokines play major role in the mechanism of cisplatin-induced cytotoxicity [10]. On the other hand, pathophysiological mechanisms of cisplatin-induced neurotoxicity include oxidative damage, inflammation, mitochondrial dysfunction, DNA damage, and apoptotic cell death in the nervous system [11, 12].

Since cisplatin-induced neurotoxicity is the major dose-limiting adverse effect of cisplatin, there are numerous studies dealing with this issue [9]. The efficacy of antioxidative treatments on preventing neurotoxicity associated with platinum-based chemotherapeutic regimens has been demonstrated in several studies [9, 13, 14]. Antioxidants such as resveratrol, curcumin, vitamin E, thiamine pyrophosphate, and melatonin have been used to reduce this type of toxicity [9, 13–17].

Oxytocin (OT) is a neurohypophyseal nonapeptide synthesized at the paraventricular and supraoptic nuclei of the hypothalamus. Although OT is essential for the milk let-down reflex, studies in OT-deficient mice show that OT's role in parturition is obviously more complex [18]. OT exerts its effects via G-protein-coupled receptors, which are expressed abundantly in the central and peripheral nervous systems. Moreover, OT plays a role in the endocrine and paracrine activities such as various sexual and maternal behaviors, social recognition, aggression, neuromodulation, cognition, and tolerance development [19]. In addition, the impact of OT on wound healing as an immunomodulatory and anti-inflammatory agent, which regulates the anti-inflammatory and proinflammatory cytokines, has also been shown [19–22].

As oxidative stress and inflammation play the major role in the pathogenesis of cisplatin-induced neurotoxicity and the antioxidant/anti-inflammatory effects of OT are well known, we hypothesized that OT may be beneficial in preventing the cisplatin-induced neurotoxicity. Therefore, we aimed to evaluate the therapeutic potential of OT in cisplatin-induced neurotoxicity by electromyography (EMG) recordings and measuring TNF-*α* levels, which is an important inflammation marker for lipid peroxidation and antioxidative capacity.

## 2. Materials and Methods

### 2.1. Animals

In this study 44 female Sprague-Dawley albino mature rats, weighing 200–220 g, were used. Animals were fed* ad libitum* and housed in pairs in steel cages, having a temperature-controlled environment (22 ± 2°C) with 12 h light/dark cycles. The experimental procedures were approved by the Animal Research Committee in Ege University. All animal studies are strictly conformed to the animal experiment guidelines of the Committee for Human Care.

### 2.2. Experimental Procedure

Of the 44 rats included, 36 were injected with a single dose of 10 mg/kg cisplatin i.p. to induce neurotoxicity development [16]. The drug-administered rats were divided into 3 groups. The first group (*n* = 12) received 1 mL/kg %0.9 NaCl (saline) i.p. for 10 days, whereas the second group (*n* = 12) and the third group (*n* = 12) were injected with 80 *μ*g/kg and 160 *μ*g/kg OT, respectively, for the same duration of time. The remaining 8 rats served as the control group and did not receive any treatment. Five rats from the saline group and 3 rats from the OT-injected groups died during the study.

### 2.3. Electrophysiological Recordings

Electromyographic (EMG) studies were performed 10 days after the injection of cisplatin. EMG recordings were obtained 3 times from the right sciatic nerve, stimulated supramaximally (intensity 10 V, duration 0.05 ms, frequency 1 Hz, in the range of 0.5–5000 Hz, 40 kHz/s with a sampling rate) by a bipolar subcutaneous needle stimulation electrode (BIOPAC Systems, Inc, Santa Barbara, CA, USA) from the sciatic notch. Compound muscle action potentials (CMAP) were recorded from 2-3 interosseous muscles by unipolar platinum electrodes. Data were evaluated by Biopac Student Lab Pro version 3.6.7 software (BIOPAC Systems, Inc) where distal latency and amplitude of CMAP were used as the parameters. During the EMG recordings, rectal temperatures of the rats were monitored by a rectal probe (HP Viridia 24-C; Hewlett-Packard Company, Palo Alto, CA, USA) and the body temperature of each rat was kept at approximately 36-37°C by a heating pad. Following the EMG recordings, animals were euthanized and blood samples were collected with cardiac puncture for biochemical measurements. These samples were centrifuged at 3000 rpm for 10 minutes at room temperature and stored at −20°C until they are assayed.

### 2.4. Measurement of Lipid Peroxidation

Lipid peroxidation was determined in the plasma samples by measuring the malondialdehyde (MDA) levels as thiobarbituric acid reactive substances (TBARS). Briefly, trichloroacetic acid and TBARS reagent were added to the plasma samples, then mixed, and incubated at 100°C for 60 min. After cooling on ice, the samples were centrifuged at 3000 rpm for 20 minutes and the absorbance of the supernatant was read at 535 nm. MDA levels were expressed as nM and tetraethoxypropane was used for calibration [23].

### 2.5. Measurement of Plasma Glutathione Levels

Glutathione (GSH) content in the plasma samples was measured spectrophotometrically according to Ellman's method [24]. In this method, thiols interact with 5,5′-dithiobis-(2-nitrobenzoic acid) (DTNB) and form a colored anion with maximum peak at 412 nm. GSH levels were calculated from the standard calibration curve and expressed as *μ*M.

### 2.6. Measurement of Plasma Tumor Necrosis Factor Alpha- (TNF-) *α* Level

Plasma TNF-*α* level was measured with commercially available enzyme-linked immunosorbent assay (ELISA) kit (Biosciences). The plasma samples were diluted 1 : 2 and TNF-*α* was determined in duplicate according to the manufacturer's guide. The detection range for the TNF-*α* assay was <2 pg/mL.

### 2.7. Statistical Analysis

Statistical analysis was performed with the Statistical Package for Social Sciences (SPSS) version 15.0 for Windows. Parametric variables were compared with Student's *t*-test and analysis of variance, whereas nonparametric variables were compared with Mann-Whitney *U* test. In addition, Shapiro-Wilk test was used for parametric-nonparametric differentiation. Results are presented as “mean ± standard error of mean (SEM).” A *P* < 0.05 was accepted as statistically significant.

## 3. Results

### 3.1. Electromyographic Results

Table 1 shows the alterations in EMG recordings in all groups. CMAP amplitude was significantly lower and the latency was significantly prolonged in the nontreated cisplatin-injected rats, compared to the control group (*P* < 0.005). When the treatment groups were compared, latency was shortened in both OT groups compared to the nontreated cisplatin-injected rats; however, the difference did not reach statistically significant level. Moreover, CMAP amplitude increased in both OT groups, compared to the nontreated cisplatin-injected rats but this increase was statistically significant only in the rats that received 160 *μ*g/kg of OT.

### 3.2. Plasma Malondialdehyde, Glutathione, and Tumor Necrosis Factor-*α* Levels

The effects of OT on plasma MDA, GSH, and TNF-*α* levels in all groups are shown in Table 2.

**Figure 1:**
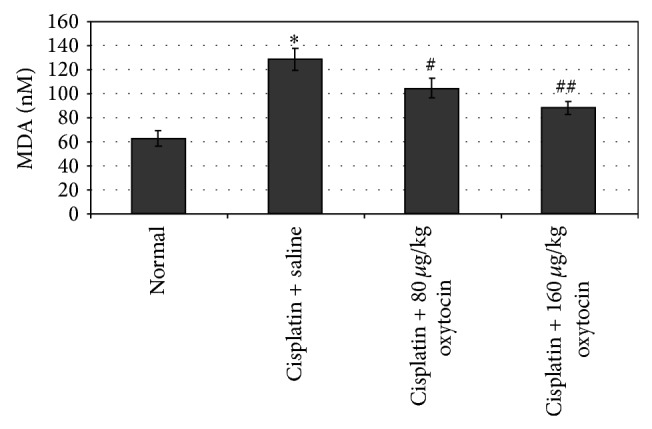
The effects of oxytocin on plasma MDA levels in all groups. Results were presented as mean ± SEM.  ^*^ *P* < 0.001, different from normal and cisplatin + saline groups;  ^#^ *P* < 0.05 and  ^##^ *P* < 0.01, different from cisplatin + saline groups.

Figure 1 shows the effects of OT on plasma MDA levels in the cisplatin-injected rats. The injection of the cisplatin resulted in a significant increase in plasma MDA levels compared to the control group (128.6 ± 9.09 versus 62.5 ± 6.08 nM; *P* < 0.001). OT treatment significantly decreased mean MDA levels in a dose-dependent manner (104.4 ± 8.11 nM for 80 *μ*g/kg OT and 88.2 ± 5.54 nM for 160 *μ*g/kg OT; *P* < 0.05 and *P* < 0.01, resp.).

**Figure 2:**
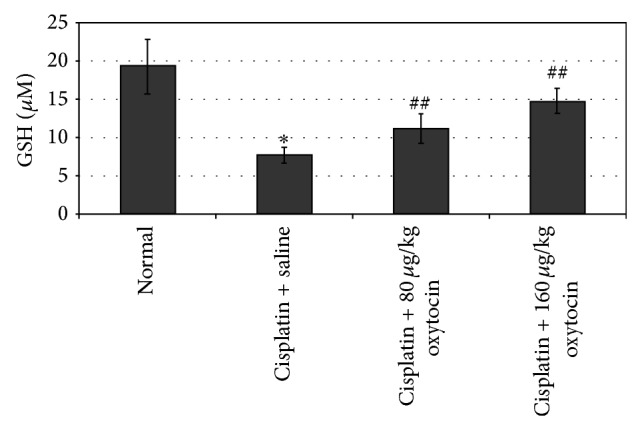
The effects of oxytocin on plasma GSH levels in all groups. Results were presented as mean ± SEM.  ^*^ *P* < 0.001, different from normal and cisplatin + saline groups;  ^##^ *P* < 0.01, different from cisplatin + saline groups.

Figure 2 demonstrates the effects of OT on plasma GSH levels in cisplatin-injected rats. Cisplatin administration significantly decreased plasma GSH levels, compared to the control group (7.78 ± 1.09 versus 19.4 ± 3.6 *μ*M; *P* < 0.001). OT treatment dose-dependently increased GSH levels (11.2 ± 1.9 *μ*M for 80 *μ*g/kg OT and 14.8 ± 1.6 *μ*M for 160 *μ*g/kg OT; *P* < 0.01).

**Figure 3:**
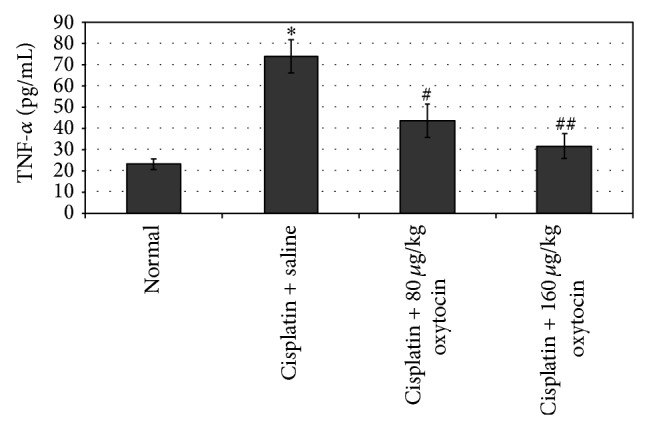
The effects of oxytocin on plasma TNF-*α* levels in all groups. Results were presented as mean ± SEM. ^*^ *P* < 0.001, different from normal and cisplatin + saline groups;  ^#^ *P* < 0.05 and  ^##^ *P* < 0.01, different from cisplatin + saline groups.

Figure 3 shows the effects of OT on plasma TNF-*α* levels in cisplatin-injected rats. Cisplatin administration significantly increased plasma TNF-*α* levels, compared to the control group (73.8 ± 7.7 versus 23.2 ± 2.6 pg/mL; *P* < 0.001). OT treatment significantly decreased TNF-*α* levels and this decrease was more evident with the increased dose of OT (43.5 ± 7.9 pg/mL for 80 *μ*g/kg OT and 31.6 ± 5.8 pg/mL for 160 *μ*g/kg OT; *P* < 0.05 and *P* < 0.01, resp.).

## 4. Discussion

In this present study, we clearly demonstrated the protective effect of OT in cisplatin-induced neurotoxicity. To our knowledge, the prevention of neurotoxic effect of cisplatin with OT treatment has been demonstrated in this study for the first time. The neuroprotective effect seems to be associated with antioxidant (by the suppression of lipid peroxidation and increasing the antioxidative capacity) and anti-inflammatory (by decreasing the plasma TNF-*α* levels) activity of OT.

There are several mechanisms proposed for cisplatin-induced neurotoxicity. The drug accumulates especially in the dorsal root ganglia and causes nucleolar damage. Moreover, it affects Schwann cells, which have an important role in the nerve development and regeneration [25]. Another explanation for the mechanism of neuropathy is the binding of the cisplatin to DNA and inhibition of DNA synthesis [26]. Oxidative stress is another important mechanism. Cisplatin increases the production of free oxygen radicals and decreases the antioxidants, thus resulting in the deterioration of the oxidant/antioxidant balance and accumulation of reactive oxygen radicals in tissues [27]. Elevated free oxygen radicals interact with DNA and result in the production of 8-hydroxyguanine (8-OH Gua), which is responsible for DNA damage [27]. Some studies also demonstrated that DNA damage and caspase activation played an essential role in cisplatin-induced toxicity [28, 29]. Furthermore, proinflammatory cytokines such as TNF-*α*, interleukin- (IL-) 1, and IL-6 are induced with the nuclear factor- (NF-) kappa B activation and result in enhanced cisplatin toxicity [30, 31]. Apoptoses induced by hypoxia, inflammation, and accumulation of reactive oxygen free radicals in tissues are also basic mechanisms which play a role in cisplatin neurotoxicity.

The imbalance between oxidative and antioxidative mechanisms may play an important role in triggering axonal injury. Axonal transport is important for axonal integrity. Excessive reactive oxygen species (ROS) production causes distal axonal degeneration and interruption of axonal transportation. ROS can directly inhibit the axonal transportation in the early phase [32]. In addition to that, adenosine triphosphate (ATP) depletion and increased intra-axonal calcium levels caused by mitochondrial damage exacerbate the axonal damage. The decrease in the ATP levels inhibits the normal functions of the motor proteins [33].

Previous clinical and experimental studies demonstrated that antioxidant agents may prevent cisplatin-induced neurotoxicity [15, 19]. OT is a secretory peptide hormone and a biochemical antioxidant. It is demonstrated that OT decreases the free oxygen radicals in the brain membranes, prevents low density lipoprotein oxidation, and inhibits lipid peroxidation [34]. When it is used at physiologic levels, OT may also decrease the acute inflammatory response, cytokine release, and oxidative stress. OT prevents lipid peroxidation on the cell membrane by scavenging the free oxygen radicals. Moreover, OT decreases lipid peroxidation, neutrophil infiltration, and serum TNF-*α* level in septic animal model [22]. Another human study demonstrated that OT decreases the levels of proinflammatory mediators such as TNF-*α*, IL-4 and 6, macrophage inflammatory proteins 1a and 1b, monocyte chemoattractant protein-1, and vascular endothelial growth factor in lipopolysaccharide-induced inflammatory response and endotoxemia [35].

Our results revealed that the protective effect of OT in cisplatin-induced neuropathy may be due to its suppression on TNF-*α* production, lipid peroxidation, and elevation of antioxidant capacity. In this study, we used pharmacologic doses of OT (80 and 160 *μ*g/kg) based on previous studies demonstrating their protective effects [36, 37]. We found that 160 *μ*g/kg oxytocin dose was more effective in the prevention of cisplatin-induced neurotoxicity.

In conclusion, this is the first study which demonstrates that OT has a protective effect in cisplatin-induced neurotoxicity via increasing the endogenous antioxidants and decreasing the lipid peroxidation and inflammation. Based on these findings, OT may be considered as a potential agent that can be used for the prevention of cisplatin-induced neurotoxicity. However, new experimental and clinical studies are required for the clinical application of this treatment modality for preventing the neurotoxicity in cancer patients.

## Figures and Tables

**Table 1:** The alterations in electromyographic recordings in all groups.

	Normal control	Cisplatin + saline	Cisplatin + 80 *μ*g/kg oxytocin	Cisplatin + 160 *μ*g/kg oxytocin
CMAP latency (ms)	2.44 ± 0.02	2.58 ± 0.02^*^	2.53 ± 0.05	2.51 ± 0.03
CMAP amplitude (mV)	10.23 ± 0.3	6.3 ± 0.26^*^	7.9 ± 0.32	8.6 ± 0.21^#^

Results were presented as mean ± standard error of mean.

^*^ *P* < 0.05 versus control group; ^#^ *P* < 0.05 versus cisplatin + saline group.

**Table 2:** The effects of oxytocin on plasma MDA, GSH, and TNF-*α* levels in all groups.

	Normal control	Cisplatin + saline	Cisplatin + 80 *μ*g/kg oxytocin	Cisplatin + 160 *μ*g/kg oxytocin
MDA (nM)	62.5 ± 6.08	128.6 ± 9.09^*^	104.4 ± 8.11^#^	88.2 ± 5.5^##^
GSH (*μ*M)	19.4 ± 3.6	7.78 ± 1.09^*^	11.2 ± 1.9^##^	14.8 ± 1.6^##^
TNF-*α* (pg/mL)	23.2 ± 2.6	73.8 ± 7.7^*^	43.5 ± 7.9^#^	31.6 ± 5.8^##^

Results were presented as mean ± SEM. ^*^ *P* < 0.001, different from normal and cisplatin + saline groups; ^#^ *P* < 0.05 and ^##^ *P* < 0.01 different from cisplatin + saline groups.
